# The influences of low protein diet on the intestinal microbiota of mice

**DOI:** 10.1038/s41598-020-74122-9

**Published:** 2020-10-13

**Authors:** Hiroaki Masuoka, Wataru Suda, Eriko Tomitsuka, Chie Shindo, Lena Takayasu, Paul Horwood, Andrew R. Greenhill, Masahira Hattori, Masahiro Umezaki, Kazuhiro Hirayama

**Affiliations:** 1 Department of Veterinary Medical Science, Graduate School of Agricultural and Life Sciences, The University of Tokyo, Tokyo, Japan; 2 RIKEN Center for Integrative Medical Sciences, Yokohama, Japan; 3 Department of Health Chemistry, Faculty of Pharmaceutical Sciences, Niigata University of Pharmacy and Applied Sciences, Niigata, Japan; 4 Department of Human Ecology, Graduate School of Medicine, The University of Tokyo, Tokyo, Japan; 5 College of Public Health, Medical and Veterinary Sciences, James Cook University, Townsville, Australia; 6 Papua New Guinea Institute of Medical Research, Goroka, Papua New Guinea; 7 School of Science, Psychology and Sport, Federation University Australia, Churchill, Australia; 8 Graduate School of Advanced Science and Engineering, Waseda University, Tokyo, Japan

**Keywords:** Symbiosis, Symbiosis, Microbiome, Microbiome

## Abstract

Recent research suggests that protein deficiency symptoms are influenced by the intestinal microbiota. We investigated the influence of low protein diet on composition of the intestinal microbiota through animal experiments. Specific pathogen-free (SPF) mice were fed one of four diets (3, 6, 9, or 12% protein) for 4 weeks (n = 5 per diet). Mice fed the 3% protein diet showed protein deficiency symptoms such as weight loss and low level of blood urea nitrogen concentration in their serum. The intestinal microbiota of mice in the 3% and 12% protein diet groups at day 0, 7, 14, 21 and 28 were investigated by 16S rRNA gene sequencing, which revealed differences in the microbiota. In the 3% protein diet group, a greater abundance of urease producing bacterial species was detected across the duration of the study. In the 12% diet protein group, increases of abundance of Streptococcaceae and Clostridiales families was detected. These results suggest that protein deficiency may be associated with shifts in intestinal microbiota.

## Introduction

Protein is an indispensable structural and functional cellular component and is essential for survival in all organisms^1^. Even when energy intake is sufficient, protein deficiency causes growth retardation and loss of muscle mass. Moreover, protein deficiency increases susceptibility to infection and induces fatty liver^2–4^, and can lead to fatigue, diarrhea, and edema^3,5^. In short, consuming a protein-deficient diet is detrimental to health and can be life-threatening^6,7^. Low protein levels can be detected by low serum albumin (Alb) and total protein (TP) concentrations, and a decrease in serum albumin-globulin (A/G) ratio^6,8^ in humans.

Recent research suggests that the onset of kwashiorkor, a disorder believed to be prevalent among children who consume a low protein diet, is related to the composition and function of the intestinal microbiota. Smith et al*.* argued that kwashiorkor is caused by a combination of “pathogenic” bacteria and a nutritionally inadequate diet in Malawi^9^. This suggests that children with “favorable” intestinal microbiota may not develop kwashiorkor even when they consume a nutritionally deficient diet. Following the same cohorts, Blanton et al*.* showed that *Ruminococcus gnavus* and *Clostridium symbiosum* ameliorated the impaired growth phenotype of children in Malawi^10^. These studies suggest that the intestinal microbiota transition during protein deficiency is associated with host homeostasis. However, the relationship between microbiota and host nitrogen balance is not fully understood. Therefore, we investigated the effects of protein deficiency on the intestinal microbiota to elucidate the potential roles of intestinal bacteria on the host nutrition.

A previous report described changes in bacterial species abundance in gnotobiotic mice inoculated with 10 gut bacterial species due to composition of diet^11^. However, few studies have investigated the response of the intestinal microbiota to a low protein diet. In this study, we investigated the influence of a low protein diet on nutritional status and intestinal microbiota composition. We fed specific pathogen-free (SPF) mice diets with different protein concentrations to investigate changes in nutritional status (i.e., food and water consumption and body weight gain), serum biomarkers (i.e., Alb, TP, A/G ratio, and blood urea nitrogen (BUN)) and the composition of intestinal microbiota between 3 and 12% protein diet groups at days 0, 7, 14, 21 and 28.

## Results

### Body weight, diet and water consumption

Body weight and food and water consumption were recorded in two independent experiments: one set up to investigate nutritional impact of protein in diet (first experiment) and the other focusing on correlations between protein content in diet and the intestinal microbiota of mice (second experiment).

**Figure 1:**
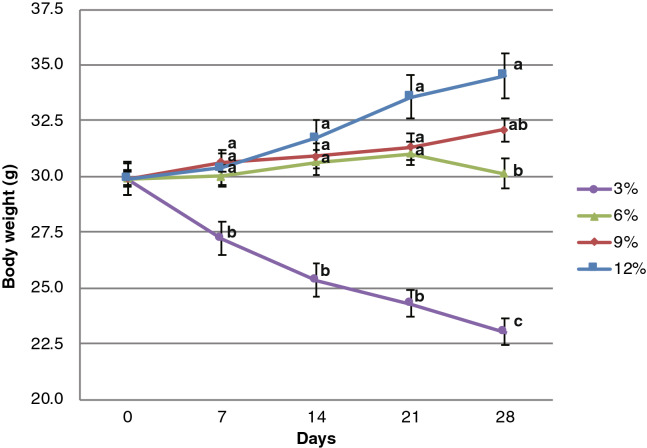
Line graph of changes in raw body weight over 4 weeks of the second experiment. Each group of mice (n = 5) was fed a diet with different protein concentration (3, 6, 9, and 12%). The mean and standard error of the mean (SEM) are shown. Plots indicated with the same letters (**a**, **b** and **c**) on the same days were not significantly different at *P* < 0.05 by Tukey–Kramer test.

A line graph of the raw body weight of each group in the first experiment can be found as Supplementary Figure S1. In the first experiment, the body weight of the 3% protein diet group was significantly lower than those of the 6% group on day 28 (*P* = 0.048 by Tukey–Kramer test), those of the 9% group from day 14 (*P* = 0.028, 0.038 and 0.013 on day14, 21 and 28, respectively, by Tukey–Kramer test) and 12% protein diet groups from day 14 (*P* = 0.0034, 0.0026 and 0.0002 on day 14, 21 and 28, respectively, by Tukey–Kramer test). There were no significant differences between the body weights of the 6%, 9% and 12% groups throughout the experimental period. The relative body weight of the 3% protein diet group decreased to 96.4 ± 2.5% on day 28, whereas the relative body weight of the 12% protein diet group increased to 120.6 ± 2.5%. A line graph of the raw body weight of each group in the second experiment can be found in Fig. 1. In the second experiment, the body weight data have similar tendency to the data of the first experiment. The body weight of the 3% protein diet group in the second experiment was significantly lower than those of the 6% group (*P* = 0.0304, 0.0002, 2.67 × 10^−5^ and 2.04 × 10^−5^ on day 7, 14, 21 and 28, respectively, by Tukey–Kramer test), 9% group (*P* = 0.0073, 0.0001, 1.52 × 10^−5^ and 9.14 × 10^−7^ on day 7, 14 ,21 and 28, respectively, by Tukey–Kramer test) and 12% protein diet groups (*P* = 0.0131, 2.30 × 10^−5^, 4.26 × 10^−7^ and 3.48 × 10^−8^ on day 7, 14, 21 and 28, respectively, by Tukey–Kramer test). There were no significant differences between the body weights of the 6%, 9% and 12% protein diet groups throughout the experimental period. The relative body weight of the 3% protein diet group decreased to 77.1 ± 2.1% on day 28, whereas the relative body weight of the 12% protein diet group increased to 115.4 ± 2.0%.

**Figure 2:**
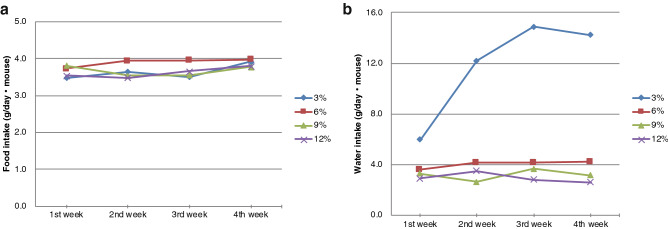
Food and water intakes of mice fed different protein diets in the second experiment. Mean values for each week are shown. “1st week” means the period from day 1 to day 7, “2nd week” means the period from day 8 to day 14, “3rd week” means the period from day 15 to day 21 and “4th week” means the period from day 22 to day 28. (**a**) Consumption of food per day (average) in mice according to protein diet. (**b**) Consumption of water per day (average) in mice according to protein diet. Differences in water and food consumption between mice fed different diets could not be investigated with statistical tests because they were evaluated by cage, thus individual variation among mice could not be evaluated.

The amount of water and food consumed by each diet group was recorded for both the nutrition and the microbiota study (Supplementary Figure S2). In the first experiment, no difference in food consumption was observed among the four groups (Figure S2a); however, water consumption was higher in the 3% protein diet group than in other groups (Figure S2b). A line graph of the amount of water and food consumption of each group in the second experiment can be found as Fig. 2. In the second experiment, food consumption was again similar across all diet groups (Fig. 2a), and water consumption was again higher in the 3% protein group (Fig. 2b). The amount of water consumed by the 3% protein diet group was more than twice that of the 12% protein diet group at day 28.

### Serum biomarkers

The bar graphs of serum concentrations can be found as Supplementary Figure S3. At the end of the experiment (day 28) the 3%, 6%, and 9% protein diet groups had significantly lower serum BUN than those in the 12% protein diet group (*P* = 1.77 × 10^−5^, 0.006 and 0.002 by Tukey–Kramer test, respectively). The serum BUN concentrations at day 28 in the 3% protein diet group were also significantly lower than that in the 6% protein diet groups (*P* = 0.034 by Tukey–Kramer test) (Figure S3a). There were no significant differences in serum concentrations of albumin, total protein and A/G at day 28 among any of the groups (Figure S3b, S3c and S3d).

### Diversity of the intestinal microbiota

**Figure 3:**
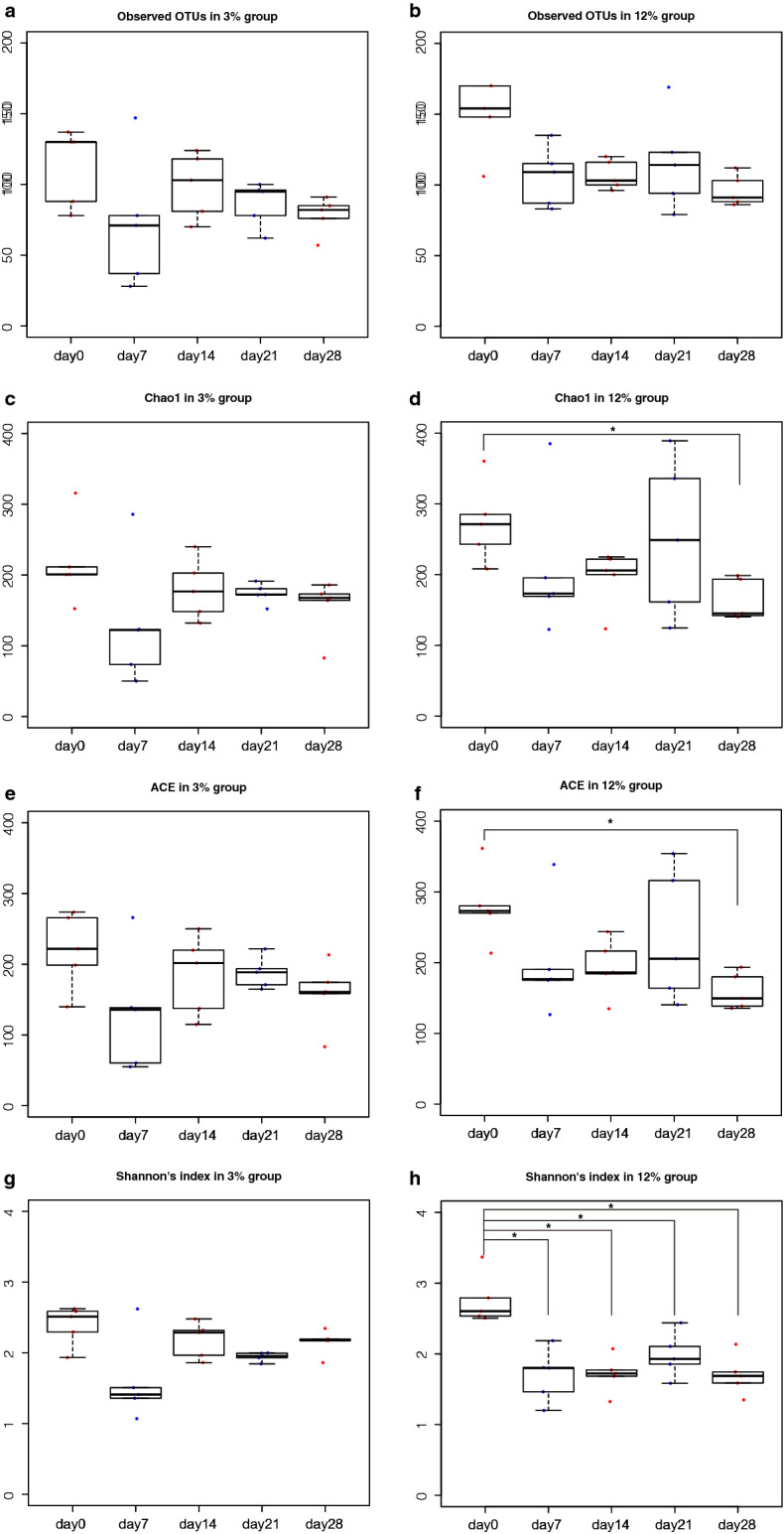
Measures of ɑ-diversity of intestinal microbiota of mice fed 3% and 12% protein diet. The label of data on day 0, 7, 14, 21 and 28, were shown as day 0, day7, day14, day21 and day28 respectively. The symbols * indicates *P* < 0.05. (**a**) The box plots of observed Operational taxonomic unit (OTU) number of the intestinal microbiota of mice in the 3% protein group. (**b**) The box plots of observed OTU number of the intestinal microbiota of mice in 12% protein group. (**c**) The box plots of Chao1 in the 3% protein group. (**d**) The box plots of Chao1 in the 12% protein group. (**e**) The box plots of ACE in the 3% protein group. (**f**) The box plots of ACE in the 12% protein group. (**g**) The box plots of Shannon’s index in the 3% protein group. (**h**) The box plots of Shannon’s index in the 12% protein group.

**Figure 4:**
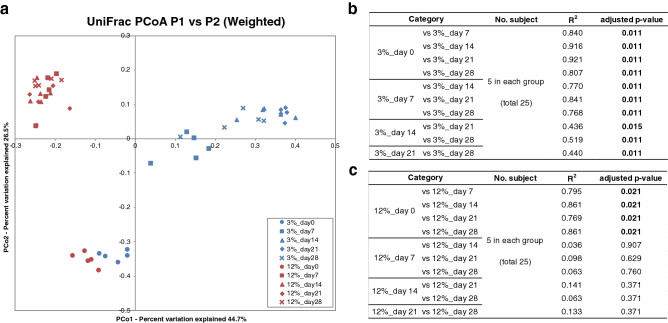
Measures of ß-diversity of intestinal microbiota of mice fed 3% and 12% protein diet. (**a**) Principal coordinates analysis (PCoA) plot based on the weighted UniFrac distances for intestinal microbiota composition in mice in each group in the second experiment. Plots of the 3% protein group were shown in blue and those of the 12% protein group were shown in red. (**b**) Evaluation of dissimilarity of the 3% protein group by PERMANOVA in the analysis of weighted UniFrac distance. R^2^ indicates coefficient of determination. Significant adjusted *P* values are in bold. (**c**) Evaluation of dissimilarity of the 12% protein group by PERMANOVA in the analysis of weighted UniFrac distance. R^2^ indicates coefficient of determination. Significant adjusted *P* values are in bold.

Figure 3 shows box plots of four α diversity indices of the intestinal microbiota in the 3% and 12% protein diets on day 0, 7, 14, 21 and 28. The OTU number, Chao1, abundance-based coverage estimator (ACE) and Shannon’s index did not differ throughout the experiment in the 3% protein diet group, whereas Chao1 and ACE were significantly lower on day 28 than on day 0 in the 12% protein diet group (*P* = 0.032 by Wilcoxon rank sum test and Benjamini–Hochberg *P *value correction). Shannon’s index in the 12% protein diet group on day 7, 14, 21and 28 were significantly lower than that on day 0. Principal coordinates analysis (PCoA) was performed on both weighted and unweighted UniFrac distances of intestinal microbiota composition of the mice in the 3% and 12% diet groups throughout the experiment (Fig. 4). Permutational multivariate analysis of variance (PERMANOVA) in the 3% protein diet group based on weighted UniFrac distance revealed that there were significant dissimilarities between day 0 and the other days (R^2^ = 0.840, *P* = 0.011 between day 0 and day 7; R^2^ = 0.916, *P* = 0.011 between day 0 and day 14; R^2^ = 0.921, *P* = 0.011 between day 0 and day 21; R^2^ = 0.807, *P* = 0.011 between day 0 and day 28). Furthermore, PERMANOVA in the 12% diet group based on weighted UniFrac distance revealed that there were also significant dissimilarities between day 0 and the other days (R^2^ = 0.795, *P* = 0.021 between day 0 and day 7; R^2^ = 0.861, *P* = 0.021 between day 0 and day 14; R^2^ = 0.769, *P* = 0.021 between day 0 and day 21; R^2^ = 0.861, *P* = 0.021 between day 0 and day 28). The dissimilarities on weighted UniFrac distances between the 3% and 12% protein diet groups at each day are shown in Supplementary Figure S4, while Supplementary Figure S5 shows a PCoA plot based on unweighted UniFrac distance.

### Compositions of the intestinal microbiota

**Figure 5:**
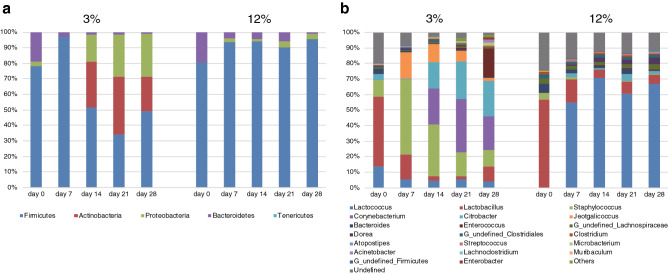
Predominant taxa by relative abundance detected in mice faeces from mice fed 3% protein diet and 12% protein diet. (**a**) Relative abundance of composition of intestinal microbiota at the phylum level across 4 weeks. (**b**) Relative abundance of composition of intestinal microbiota at the genus level across 4 weeks.

Microbial composition analyses were conducted at phylum level for phyla present at an abundance > 0.1% in at least one diet group and compared for different time points (Fig. 5a). Outcomes of statistical analyses, using Wilcoxon rank sum test with Benjamini–Hochberg *P* value procedure, are provided in Supplementary Table S1a and S1b, with a summary of key findings provided below. In the 3% protein diet group, the proportions of Actinobacteria were significantly higher on day 14, 21 and 28 than on day 0 (*P* = 0.032, 0.032 and 0.032, respectively). The proportions of Bacteroidetes were significantly lower on day 7, 14, 21 and 28 than on day 0 (*P* = 0.032, 0.032, 0.032 and 0.032, respectively). The proportions of Firmicutes were significantly higher on day 7 than on day 0 (*P* = 0.032), whereas those on day 14, 21 and 28 were significantly lower than on day 0 (*P* = 0.032, 0.032 and 0.032, respectively). The proportions of Proteobacteria were significantly higher on day 14, 21 and 28 than on day 0 (*P* = 0.048, 0.032 and 0.048, respectively). The proportions of Tenericutes were significantly lower on day 28 than on day 0 (*P* = 0.032). On the other hand, in the 12% protein diet group, the proportions of Bacteroidetes were significantly lower on day 14 and 21 than on day 0 (*P* = 0.032 and 0.032, respectively). The proportions of Firmicutes were significantly higher on day 14 and 21 than on day 0 (*P* = 0.032 and 0.032, respectively). The proportions of Proteobacteria were significantly higher on day 7, 14, 21 and 28 than on day 0 (*P* = 0.048, 0.032, 0.032 and 0.032, respectively). The proportions of Actinobacteria were not significantly changed throughout the experiment. In addition to *P* values, Tables S1a and 1b provide average read numbers in each group throughout the experiment for these phyla.

Analysis was also conducted at genus level for genera with > 0.1% abundance in at least one diet group (Fig. 5b). In the 3% protein diet group, there were bacteria whose relative abundance increased or decreased throughout the experiment. Outcomes of statistical analyses, using Wilcoxon rank sum test with Benjamini–Hochberg *P *value procedure are provided in Supplementary Table S1c, and summarized below. The proportions of *Acinetobacter*, *Enterobacter* and *Enterococcus* were significantly higher on day 28 than those on day 0 (*P* = 0.032 for all three genera). The proportions of *Microbacterium* were significantly higher on day 21 and 28 than that on day 0 (*P* = 0.032 and 0.032). The proportions of *Corynebacterium* were significantly higher on day 14, 21 and 28 than that on day 0 (*P* = 0.032, 0.032 and 0.032). The proportions of *Jeotgalicoccus* were significantly higher on days 7, 14, 21 and 28 than those on day 0 (*P* = 0.032, 0.032, 0.032 and 0.032). The proportions of *Staphylococcus* were significantly higher on day 7 than that on day 0 (*P* = 0.032). The proportions of *Citrobacter* were significantly higher on day 14 and 21 than that on day 0 (*P* = 0.048 and 0.032, respectively). The proportions of *Lactobacillus*, *Lactococcus* and *Muribaculum* were significantly higher on day 0 than those on days 7, 14, 21 and 28 (*P* = 0.032, 0.032, 0.032 and 0.032 for all three genera) and the proportions of *Bacteroides* also changed across the four sample days (*P* = 0.048, 0.048, 0.032 and 0.048, respectively). The proportions of *Dorea* were significantly higher on day 0 than those on day 14, 21 and 28 (*P* = 0.048, 0.048 and 0.032, respectively). In the 12% protein diet group, there were also bacteria whose relative abundance increased or decreased throughout the experiment. The proportions of *Dorea*, *Lactococcus* and *Streptococcus* were significantly lower on day 0 than on days 7, 14, 21 and 28 (*P* = 0.032, 0.032, 0.032 and 0.032 for all four genera). The proportions of *Citrobacter* (*P* = 0.048, 0.032, 0.032 and 0.032, respectively) and *Enterococcus* (*P* = 0.048, 0.048, 0.048 and 0.032, respectively) changed in the same way over days 7, 14, 21 and 28. The proportions of *Lactobacillus*, *Muribaculum* and *Staphylococcus* were significantly higher on day 0 than on days 7, 14, 21 and 28 (*P* = 0.032, 0.032, 0.032 and 0.032 for all three genera). The proportions of *Atopostipes* were significantly higher on day 0 than that on day 21 and 28 (*P* = 0.048 and 0.032, respectively). The proportions of *Bacteroides* were significantly higher on day 0 than that on day 28 (*P* = 0.032). Average read numbers in each group and *P* values between day 0 and the other each day of these genera can be found in Supplementary Table S1c and 1d.

**Figure 6:**
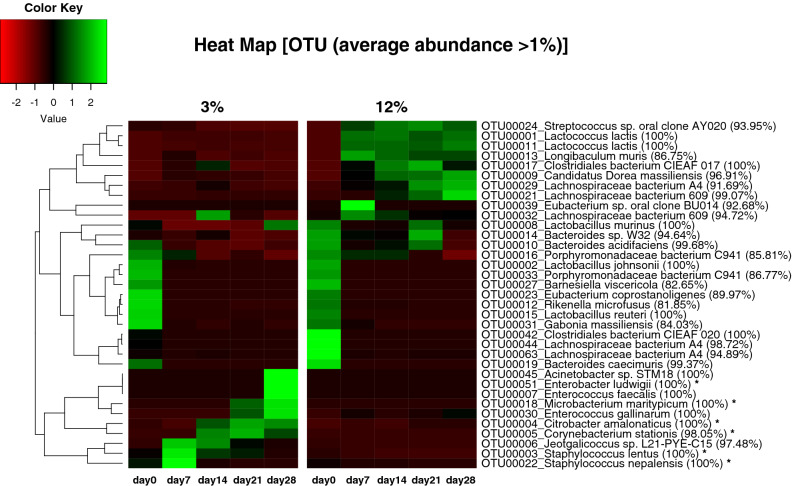
Heatmap of the phylotypes assigned to species-level taxa for OTUs with more than 1% abundance in at least 1 group. The color in the heatmap reflects the z-score; green shows that the ratio of OTUs are high in each group, and red shows that that are low in each group. The symbols * indicates OTUs assigned to urease-positive bacteria.

Figure 6 provides a heatmap which represents abundance of the selected OTUs with more than 1% abundance in at least one diet group average from day 0 to day 28 in either groups. The read numbers of OTUs shown in Fig. 6 and their *P* values between day 0 and the other each day in each group can be found as Supplementary Table S2. The total average abundance of these selected OTUs accounted for 90.4 ± 1.10%.

## Discussion

In the present study, we investigated the effects of low protein diet on the composition of intestinal microbiota and nutritional status in male SPF mice. We used only male mice to remove sex-related effects thus ensuring the analysis was focused on the intestinal microbiota change related to differences in nutrition. Mice fed a 3% protein diet showed signs of protein deficiency, such as growth retardation and low serum BUN. The consistent food intake throughout the experiment suggests that weight loss observed in the 3% protein group was not caused by a decrease in the amount of food consumed, as the amount of food consumed did not differ amongst the four dietary groups. Interestingly, the amount of water consumption by the 3% protein group was considerably higher than that consumed by the other groups. Other researchers have investigated food intake in low-protein diets, but they provided inconsistent results. Our results on food consumption corresponded with the findings of Larson et al.^12^, but are contrary to the other research in which an increase in food consumption was reported^13,14^. Inconsistent results across numerous studies are difficult to fully explain, but may reflect differences in age, sex and breed. In the current study, we demonstrated that mice fed a low protein intake consumed more water. To our knowledge, this is the first report of protein intake correlating with water intake.

Previous reports indicated that the plasma albumin level of rats tended to decrease at some time points when they were fed low protein diet^15,16^. On the other hand, Wada et al*.* indicated that there was no difference in plasma albumin between control groups and low protein groups after 4 weeks^16^. Hubert et al. also revealed that there was almost no difference in plasma albumin between ad libitum group and diet-restricted group after 4 weeks^17^. In agreement with these previous studies, we also showed that plasma albumin concentration among mice fed a low protein diet did not decrease over the 4 weeks of our study.

In the present study, there were differences in gut microbial composition between the 3% and 12% groups at day 0, yet the dissimilarity on UniFrac distances between the 3% and 12% protein diet groups at day 0 were significantly lower than those of the other time points (Supplementary Figure S4). Thus, it is regarded that the variations detected in this study were mostly affected by dietary changes, though the difference at day 0 might be related to cage-specific effects. These microbiota shifts accompanied by dietary changes potentially include not only differences in nutrition but also host systemic changes such as weight loss. In reaction to the 3% protein diet, the proportions of 10 predominant OTUs assigned to *Staphylococcus lentus*, *Citrobacter amalonaticus*, *Enterococcus faecalis*, *Jeotgalicoccus* sp., *Corynebacterium stationis*, *Staphylococcus nepalensis*, *Enterobacter ludwigii*, *Acinetobacter* sp*.*, *Enterococcus gallinarum* and *Microbacterium maritypicum* increased. Among these OTUs, 6 OTUs including *E. ludwigii*, *M. maritypicum*, *C. amalonaticus*, *C. stationis*, *S. lentus* and *S. nepalensis* have been previously reported to have ureases, the enzymes catalyzing the hydrolysis of urea into carbon dioxide and ammonia^18–23^. It was reported that urea in the gastrointestinal tract excreted from epithelial cells is cleaved into ammonia and carbon dioxide when sufficient urease is produced by intestinal bacteria^24^, and some bacteria utilize ammonia to produce amino acids and peptides^25^. The host can absorb either the amino acids or the peptides synthesized by the bacteria. This process is called “urea nitrogen salvage” caused by urease-producing and ammonia-utilizing bacteria. It is also known that the absorption of ammonia produced from urea and its recycling into amino acids occur at the host’s systemic levels^26^. Further studies would be necessary to clarify the contribution of microbiota to host’s nitrogen balances.

In reaction to the 12% protein diet, the proportion of 8 predominant OTUs assigned to *Streptococcus* sp., *Lactococcus lactis* (2 OTUs), *Longibaculum muris*, Clostridiales bacterium, Candidatus *Dorea massiliensis* and Lachnospiraceae bacterium (2 OTUs) increased. Among these 8 OTUs, 3 OTUs assigned to *L. lactis* and *Streptococcus* sp. belong to the Streptococcaceae family, and the other 5 out of 8 OTUs assigned to the Clostridiales bacterium, Candidatus *Dorea massiliensis* and Lachnospiraceae bacterium belong to the Lachnospiraceae family. OTUs assigned to Streptococcaceae family increased even in the 3% protein group, but showed significant increase in the 12% protein group. The high proportion of *L. lactis* in the 12% protein diet group may have been due to composition of the diet. The protein sources of the NMF diet used for acclimatization are plant and animal based, i.e., soybeans and fish powders; whereas those of the 3% and 12% protein diets are caseins (from mammalian milk). A previous report by Bisanz et al.^27^ suggested that *Lactococcus* species might be favored by casein in experimental diets.

Several limitations should be discussed. First, we did not set a control group by feeding commercial solid diet (containing 27.5% of protein), although that was used for acclimatization. Since our purpose was to compare the impact of “a diet containing a sufficient amount of protein” and “protein deficient diets”, we judged that a commercial diet contains an excess amount of protein, relative to the needs of mice. Thus, the 12% protein diet more appropriately represents “a diet containing a sufficient amount of protein”. Secondly, differences in intestinal microbiota between the 3% and 12% group at baseline were observed; we sought to randomize individual mice so average body weight became comparable under each diet. The reason of differences at the baseline could be that intestinal microbiota changed non-directionally during this period in mice of each cage. To confirm if this change in intestinal microbiota during the acclimatization period resulted in discordance in intestinal microbiota at day 0 of experiment among cages, we calculated weighted UniFrac distance at baseline among mice of 3% protein group (n = 5), among mice of 12% protein group (n = 5) and among mice of 3% and 12% protein groups together (n = 10) (Supplementary Figure S6). It was revealed that discordance in intestinal microbiota observed in mice of 3% and 12% protein groups together did not differ from that observed in mice of 3% protein or 12% protein group only. In other words, differences in gut microbiota between 3% protein group and 12% group at baseline are comparable to that observed in each group.

Intestinal microbiota and their hosts are in a symbiotic relationship. Previous studies indicated that the changes in nutritional composition of the diet can affect host health via intestinal microbiota shifts^28,29^. In this study, we detected characteristic changes in intestinal microbiota in the 3% and 12% protein groups. Specifically, in the 3% group we detected the increase of abundance of several bacterial species suggested to be related to “urea nitrogen salvage”. Further studies to clarify the roles of intestinal microbiota related to protein intake will contribute to solving challenges associated with inadequate human nutrition, particularly in low-income countries.

## Methods

### Animals

Male BALB/cAJcl SPF mice (9 weeks of age) were purchased from CLEA Japan Inc. (Tokyo, Japan). The mice were kept in a room at 24 ± 1 °C, 55 ± 5% relative humidity, on a 12-h light/dark cycle. The animals were housed in cages (n = 5 per cage) with wood shavings and fed and watered ad libitum. All experiments were approved by the Animal Care and Use Committee of the University of Tokyo. All methods were conducted in strict accordance with the guidelines regarding animal research of the University of Tokyo.

### Experimental design

In the first experiment, all mice (n = 20) were acclimatized on a commercial solid diet (NMF; Oriental Yeast Co, Ltd., Tokyo, Japan) for 1 week. After acclimatization, we randomized mice individuals based on body weight and then divided mice into four groups and fed them a diet containing 3, 6, 9, or 12 g of protein/100 g dry weight (Oriental Yeast Co., Ltd). The nutritional components of each diet are shown in Supplementary Table S3. These four groups were fed in parallel. Mice were individually recognized using a picric acid-saturated solution with ethanol. Protein concentrations of experimental diet were determined based on an experiment by Nakata et al*.* that demonstrated lower body weight gain with a diet containing 8% protein compared to that with a 12% protein diet^30,31^. Body weight was individually measured weekly, and food and water consumption were measured weekly per group. After the feeding experiment for 4 weeks, the mice were euthanized using carbon dioxide. Whole blood samples were collected during exsanguination on experimental day 28, centrifuged for 15 min at 1500×g, and the serum samples were stored at − 80 °C until analysis.

In the second experiment, an additional 20 mice were acclimatized, randomized and divided into four groups as occurred in the first experiment. Body weight was individually measured weekly, and food and water consumption were measured weekly per group. After the feeding experiment for 4 weeks, the mice were euthanized using carbon dioxide. Fecal samples were collected from each mouse in the 3% and 12% protein diet groups directly at defecation every seven days (day 0, 7, 14, 21 and 28), and then stored at − 80 °C until 16S rRNA gene sequencing was conducted.

### Sample size calculation

For estimating minimum sample size for detecting difference in body weight on day 28, we analyzed the results of the first experiment which used 20 mice (n = 5 mice per group) in total. We calculated a minimum sample size for the second experiment by assuming significance level 0.05 and statistical power 0.80. Minimum sample sizes on day 14, 21 and 28 were estimated to be 16, 16 and 15 in total, respectively (Supplementary Table S4). Based on this result, we decided to allocate 5 mice in each cage and feed different protein diet.

### Serum biomarker analysis

Serum Alb concentrations were measured using the BCG method, and serum TP concentrations were measured using the biuret method following the manufacturer’s instructions (A/G B-test Wako, Wako Pure Chemical, Osaka, Japan). The serum A/G ratio was calculated as Alb/(TP − Alb). BUN was measured using a urease-indophenol method (urea nitrogen-B test; Wako Pure Chemical).

### Bacterial DNA extraction

16S rRNA gene sequencing analysis was performed using fecal samples from the 3% and 12% protein diet groups at day 0, 7, 14, 21 and 28. Bacterial DNA was extracted following a previously described method^32^. Frozen fecal pellets were thawed and suspended in 1 mL of TE10 (10 mM Tris–HCl, 10 mM EDTA) buffer containing RNaseA (final concentration of 100 µg/mL, NIPPON GENE, Tokyo, Japan). Lysozyme (Sigma-Aldrich Co., Tokyo, Japan) was added to a final concentration of 15 mg/mL, and the suspension was incubated for 1 h at 37 °C with gentle mixing. Purified achromopeptidase (Wako Pure Chemical) was added to a final concentration of 2000 units/mL, and the suspension was further incubated for 30 min at 37 °C. Sodium dodecyl sulfate (final concentration 1 mg/mL) and proteinase K (final concentration 1 mg/mL, Merck Japan, Tokyo, Japan) were added to the sample and this mixture was incubated for 1 h at 55 °C. The DNA was extracted with phenol/chloroform/isoamylalcohol (25:24:1), precipitated with isopropanol and 3 M sodium acetate, washed with 75% ethanol, and resuspended in 200 µL of TE buffer. The DNA was purified with a 20% PEG solution (PEG6000 in 2.5 M NaCl), pelleted by centrifugation, rinsed with 75% ethanol, and dissolved in TE buffer.

### 16S rRNA gene amplicon sequencing

The V1-V2 region of the bacterial 16S rRNA gene was amplified by polymerase chain reaction (PCR) using the universal primers 27Fmod (5′-AGRGTTTGATYMTGGCTCAG-3′) and 338R (5′-TGCTGCCTCCCGTAGGAGT-3′) as described previously^33^. Amplicons generated from each sample (~ 330 bp) were subsequently purified using AMPure XP (Beckman Coulter). DNA was quantified using a Quant-iT Picogreen dsDNA assay kit (Invitrogen) and a TBS-380 Mini-Fluorometer (Turner Biosystems). The 16S rRNA gene sequencing was performed with the MiSeq sequencing system (Illumina, San Diego, CA, USA) according to Illumina protocol.

### Data analysis

Two paired-end reads were merged using the fastq-join program. The reads lacking both forward and reverse primer sequences were removed using blast followed by trimming off both primer sequences. The reads with average quality values < 25 were further removed. Among these high-quality reads, 3000 reads/sample were randomly selected and were subjected to operational taxonomic unit clustering and UniFrac analysis. The selected reads of all the samples were firstly sorted by frequency of redundant sequences and grouped into operational taxonomic units (OTUs) using UCLUST (https://www.drive5.com/) with a sequence identity threshold of 97%. The sequence with the highest redundancy in each OTU was determined as a representative sequence. The representative sequences of the OTUs were subjected to homology search against the following 16S databases using the GLSEARCH program for taxonomic assignments. For the assignment at the phylum, genus, and species levels, sequence similarity thresholds of 70%, 94% and 97%, respectively, were applied. The 16S database was reconstructed from three publicly available databases: Ribosomal Database Project (RDP) v. 10.27, CORE (https://microbiome.osu.edu/), and a reference genome sequence database obtained from the NCBI FTP site (ftp://ftp.ncbi.nih.gov/genbank/, December 2011).

The richness of the microbiota was estimated by the Chao1 and ACE indices^34,35^. Shannon’s index was calculated to assess diversity^36^. PCoA was performed based on UniFrac distances.

### Statistical analysis

All statistical analyses were conducted with the R software program (v3.5.1). Body weight measured during the experiment was expressed as raw body weight. Impact of protein content in diet on body weight and serum biomarkers were investigated by using one-way ANOVA and Tukey–Kramer post hoc tests. Difference in water and food consumption by type of diet could not be tested statistically because they were measured by cage and individual variation among mice in a cage were not available.

Wilcoxon rank sum test was used for comparison of alpha diversities and the proportion of intestinal bacteria at the phylum, genus, and OTU levels. PERMANOVA was used to compare the intestinal microbiota structure among groups based on both weighted and unweighted UniFrac distance^37^. *P* values were corrected for multiple testing using the Benjamini–Hochberg method as appropriate.

## Supplementary information

Supplementary information.

## Data Availability

All data generated or analyzed during this study are included in this published article and its supplementary information files. The output data generated by next-generation sequencing in this paper have been submitted to the DDBJ Sequence Read Archive (DRA) under the accession number DRA009131 and DRA009688.
