# Supplementary material for: The influences of low protein diet on the intestinal microbiota of mice
Source: Sci Rep. 2020 Oct 13;10:17077. doi: 10.1038/s41598-020-74122-9 (PMC7555506; doi:10.1038/s41598-020-74122-9)
Supplement: Supplementary file 1 — Supplementary information. [file 41598_2020_74122_MOESM1_ESM.pdf]

The influences of low protein diet on the intestinal microbiota of mice

Hiroaki Masuoka[1*], Wataru Suda[2*], Eriko Tomitsuka[3], Chie Shindo[2], Lena Takayasu[2,4], Paul Horwood[5,6], Andrew R. Greenhill[6,7], Masahira Hattori[2,8], Masahiro Umezaki[4], and Kazuhiro Hirayama[1]

[1] Department of Veterinary Medical Science, Graduate School of Agricultural and Life Sciences, The University of Tokyo, Tokyo, Japan

[2] RIKEN Center for Integrative Medical Sciences, Yokohama, Japan

[3] Department of Health Chemistry, Faculty of Pharmaceutical Sciences, Niigata University of Pharmacy and Applied Sciences, Niigata, Japan

[4] Department of Human Ecology, Graduate School of Medicine, The University of Tokyo, Tokyo, Japan

[5] College of Public Health, Medical and Veterinary Sciences, James Cook University, Townsville, Australia

[6] Papua New Guinea Institute of Medical Research, Goroka, Papua New Guinea

[7] School of Science, Psychology and Sport, Federation University Australia, Churchill, Australia

[8] Graduate School of Advanced Science and Engineering, Waseda University, Tokyo, Japan

[*] hiroaki-masuoka@g.ecc.u-tokyo.ac.jp

[*] wataru.suda@riken.jp

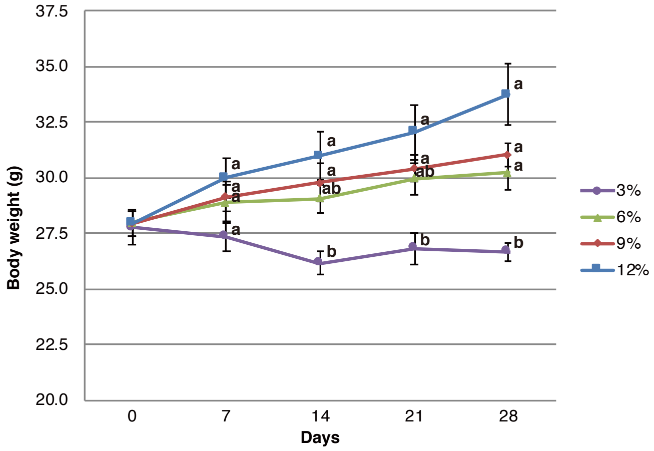

Supplementary Figure S1. Changes in raw body weight in over 4 weeks in the first experiment. Each group of mice (n=5) was fed a diet with different protein concentration (3, 6, 9, and 12%). The mean and standard error of the mean (SEM) are shown. Plots indicated with the same letters (**a**, **b** and **c**) on the same days were not significantly different at P <0.05 by Tukey-Kramer test.

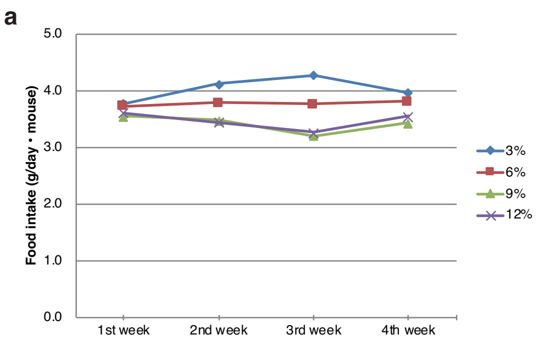

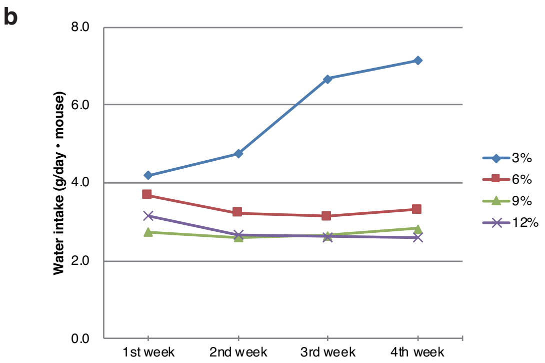

Supplementary Figure S2. Food and water intakes of mice fed different protein diets in the first experiment. Mean values for each week are shown. "1st week" means the period from day 1 to day 7, "2nd week" means the period from day 8 to day 14, "3rd week" means the period from day 15 to day 21 and "4th week" means the period from day 22 to day 28. **(a)** Consumption of food per day (average) in mice according to protein diet. **(b)** Consumption of water per day (average) in mice according to protein diet.

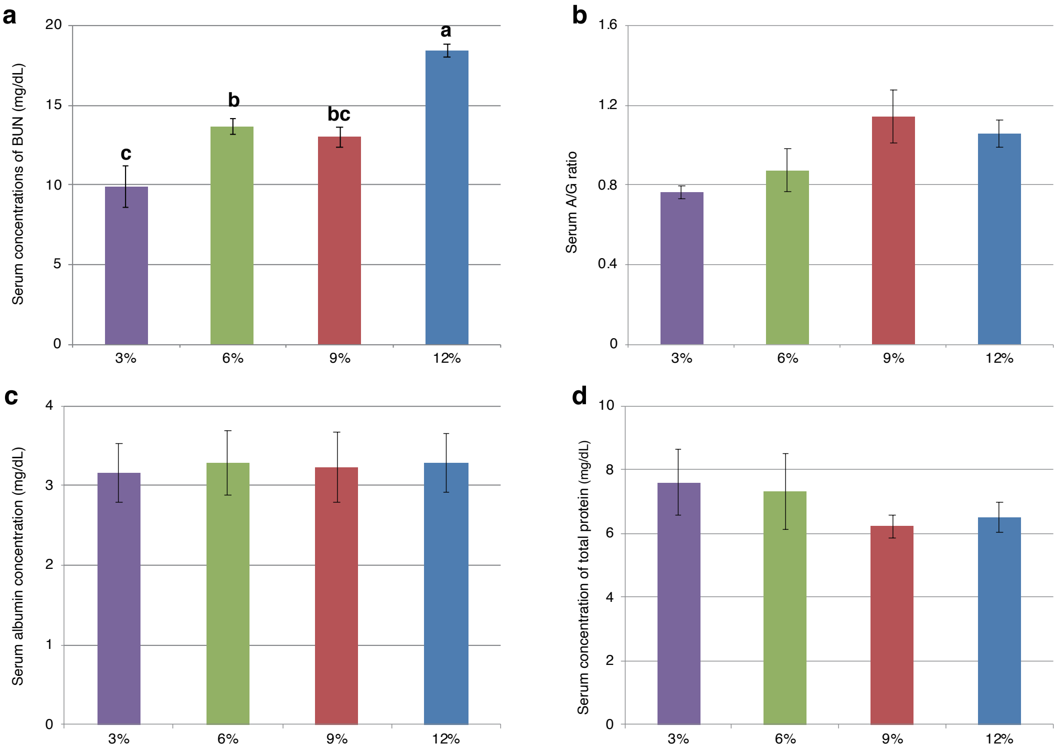

Supplementary Figure S3. Nutritional levels from blood analysis of mice fed differing protein diets in the first experiment. The mean and standard error of the mean (SEM) are shown. Bars in **a** indicated with the same letters (a, b and c) on the same days were not significantly different at P <0.05 by Tukey-Kramer test. There were no differences between each group in **b, c** and **d**. **(a)** Serum concentration of urea nitrogen (UN). **(b)** Serum albumin-globulin (A/G) ratio. **(c)** Serum albumin concentration. **(d)** Serum concentration of total protein.

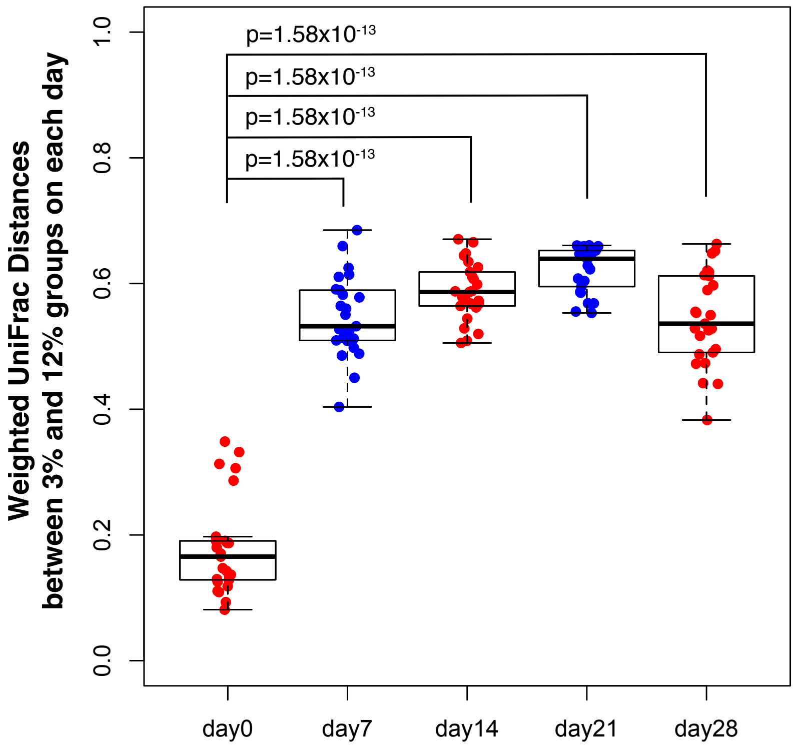

Supplementary Figure S4. Weighted UniFrac distances between 3% and 12% diet groups on each day. The label of data on day 0, 7, 14, 21 and 28, were shown as day 0, day 7, day 14, day 21 and day 28 respectively.

a

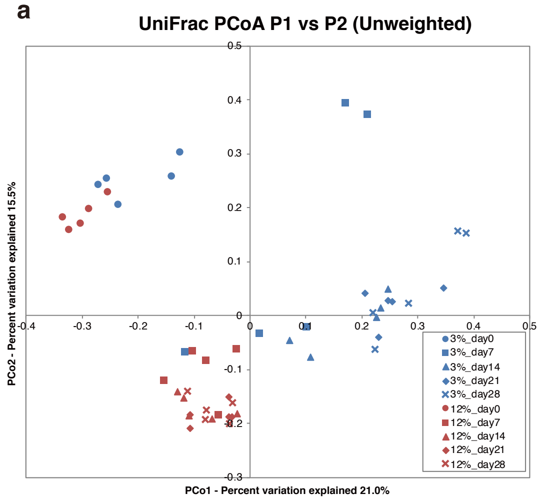

b

Category		No. subject	R^2	adjusted p-value
3%_day 0	vs 3%_day 7	5 in each group (total 25)	0.297	**0.009**
	vs 3%_day 14		0.460	**0.009**
	vs 3%_day 21		0.487	**0.009**
	vs 3%_day 28		0.524	**0.009**
3%_day 7	vs 3%_day 14		0.222	**0.009**
	vs 3%_day 21		0.260	**0.009**
	vs 3%_day 28		0.294	**0.009**
3%_day 14	vs 3%_day 21		0.212	**0.009**
	vs 3%_day 28		0.314	**0.009**
3%_day 21	vs 3%_day 28		0.223	**0.009**

c

Category		No. subject	R^2	adjusted p-value
12%_day 0	vs 12%_day 7	5 in each group (total 25)	0.409	**0.015**
	vs 12%_day 14		0.452	**0.015**
	vs 12%_day 21		0.455	**0.015**
	vs 12%_day 28		0.471	**0.015**
12%_day 7	vs 12%_day 14		0.141	0.057
	vs 12%_day 21		0.170	**0.015**
	vs 12%_day 28		0.172	**0.015**
12%_day 14	vs 12%_day 21		0.102	0.605
	vs 12%_day 28		0.169	**0.015**
12%_day 21	vs 12%_day 28		0.160	0.055

Supplementary Figure S5. (**a**) Principal coordinates analysis (PCoA) plot based on the unweighted UniFrac distances for intestinal microbiota composition in mice in each group in the second experiment. Plots of the 3% protein group were shown in blue and those of the 12% protein

group were shown in red. (**b**) Evaluation of dissimilarity of the 3% protein group by PERMANOVA in the analysis of unweighted UniFrac distance. R^2 indicates coefficient of determination. Significant adjusted p values are in bold. (**c**) Evaluation of dissimilarity of the 12% protein group by PERMANOVA in the analysis of unweighted UniFrac distance. R^2 indicates coefficient of determination. Significant adjusted P values are in bold.

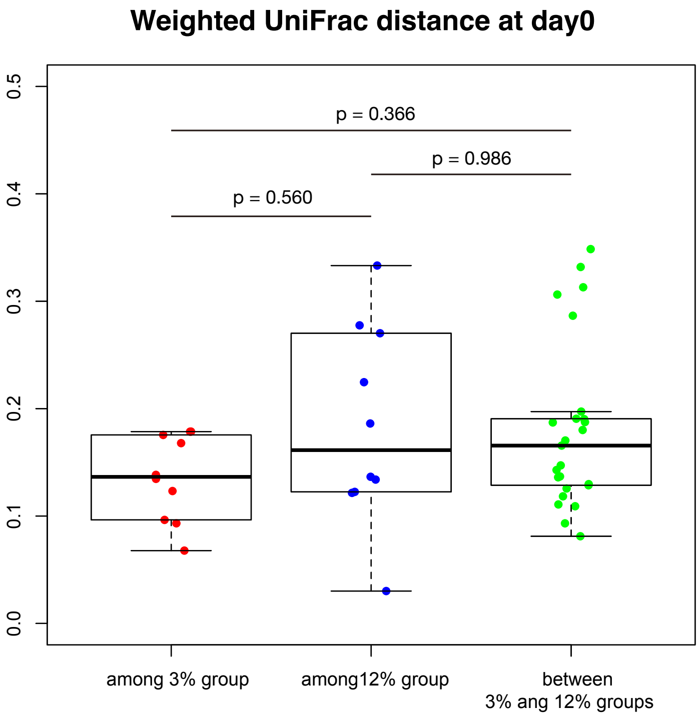

Supplementary Figure S6. Comparison between weighted UniFrac distance in intestinal microbiota at baseline among mice of 3% protein group (red), among mice of 12% protein group (blue) and among mice of 3% and 12% protein groups together (green).

Supplementary Table S1. (a) The list of taxa in the 3% protein group at phylum level with more than 0.1% abundance in at least one group. (b) The list of taxa in the 12% protein group at phylum level with more than 0.1% abundance in at least one group. **(c)** The list of taxa in the 3% protein group at genus level with more than 0.1% abundance in at least one group average. **(d)** The list of taxa in the 12% protein group at genus level with more than 0.1% abundance in at least one group.

(a)

Phylum in 3%	day 0			day 7			day 14			day 21			day 28			Adjusted p values day 0 vs day 7	vs day 14	vs day 21	vs day 28
Firmicutes	2342.8	±	73.5	2906.8	±	73.8	1553.2	±	125.2	1023	±	105.1	1474.4	±	278.0	**0.032**	**0.032**	**0.032**	**0.032**
Actinobacteria	0.2	±	0.2	0.4	±	0.4	875.8	±	27.0	1124.6	±	40.2	675.8	±	67.7	1.000	**0.032**	**0.032**	**0.032**
Proteobacteria	83.4	±	75.8	9.2	±	4.5	526.4	±	140.1	804.4	±	121.0	821.2	±	254.9	0.825	**0.048**	**0.032**	**0.048**
Bacteroidetes	569.6	±	47.0	82.6	±	71.2	44.2	±	20.1	46.8	±	25.6	28.4	±	22.2	**0.032**	**0.032**	**0.032**	**0.032**
Tenericutes	3.0	±	0.7	1.0	±	0.5	0.4	±	0.2	1.2	±	0.2	0	±	0.0	0.159	0.071	0.159	**0.032**

(b)

Phylum in 12%	day 0			day 7			day 14			day 21			day 28			Adjusted p values day 0 vs day 7	Adjusted p values day 0 vs day 14	Adjusted p values day 0 vs day 21	Adjusted p values day 0 vs day 28
Firmicutes	2417.8	±	116.7	2809.4	±	67.9	2828.2	±	43.9	2702.8	±	98.3	2866.4	±	36.9	0.063	**0.032**	0.151	**0.032**
Proteobacteria	0.0	±	0.0	68.4	±	52.2	41.2	±	19.2	114.2	±	45.8	96.6	±	35.9	**0.048**	**0.032**	**0.032**	**0.032**
Bacteroidetes	578.4	±	116.9	119.8	±	70.5	129.4	±	33.1	178.8	±	90.2	32.6	±	6.4	0.063	**0.032**	0.063	**0.032**

(c)

Genus in 3% group	day 0	day 7	day 14	day 21	day 28	Adjusted p values day 0			
						vs day 7	vs day 14	vs day 21	vs day 28
Staphylococcus	314.6 ± 100.5	1465.4 ± 186.1	1011.4 ± 49.1	460.4 ± 138.5	319.2 ± 17.1	**0.032**	0.167	0.841	0.841
Lactobacillus	1356.4 ± 133.4	475.4 ± 49.0	82.6 ± 16.9	60 ± 27.4	287.8 ± 112.4	**0.032**	**0.032**	**0.032**	**0.032**
Corynebacterium	0 ± 0.0	0.4 ± 0.4	686.2 ± 25.7	1033 ± 33.1	661.2 ± 61.3	1.000	**0.032**	**0.032**	**0.032**
Citrobacter	119.4 ± 75.8	14.4 ± 4.2	515 ± 139.7	720.2 ± 121.1	675.2 ± 213.9	0.825	**0.048**	**0.032**	0.063
Enterococcus	6.4 ± 0.4	0.4 ± 0.2	0 ± 0.2	29 ± 21.4	553 ± 197.9	0.810	0.810	0.381	**0.032**
Jeotgalicoccus	3 ± 0.7	495.4 ± 183.0	347 ± 26.5	211.8 ± 16.0	59.6 ± 14.1	**0.032**	**0.032**	**0.032**	**0.032**
Lactococcus	405 ± 0.0	158.6 ± 54.1	134.2 ± 30.7	165.4 ± 9.8	117.6 ± 11.6	**0.032**	**0.032**	**0.032**	**0.032**
Bacteroides	98 ± 18.7	34.2 ± 20.2	9.8 ± 11.7	23.8 ± 5.8	21 ± 18.6	**0.048**	**0.048**	**0.032**	**0.048**
Microbacterium	0 ± 0.0	0 ± 0.0	0 ± 0.0	32.4 ± 7.7	66.4 ± 12.2	1.000	1.000	**0.032**	**0.032**
Acinetobacter	0 ± 0.0	0 ± 0.0	0 ± 0.0	0 ± 0.0	55.4 ± 20.0	1.000	1.000	1.000	**0.032**
Enterobacter	0 ± 0.0	0 ± 0.0	0 ± 0.0	0 ± 0.0	36.6 ± 23.5	1.000	1.000	1.000	**0.032**
Dorea	18 ± 0.2	21.6 ± 20.2	8.8 ± 6.7	15 ± 3.9	9.6 ± 1.4	0.286	**0.048**	**0.048**	**0.032**
Muribaculum	15.8 ± 3.2	5 ± 0.0	0.4 ± 0.5	1.8 ± 0.9	0.4 ± 0.0	**0.032**	**0.032**	**0.032**	**0.032**
Lysinibacillus	0 ± 0.0	0 ± 0.0	0.8 ± 1.0	13.6 ± 5.3	4.2 ± 2.3	1.000	0.095	**0.032**	**0.032**
Pseudoclavibacter	0 ± 0.0	0 ± 0.0	0 ± 0.0	1.2 ± 0.7	12.4 ± 2.8	1.000	1.000	0.143	**0.032**
Bacillus	0 ± 0.0	0 ± 0.0	0.2 ± 0.8	9 ± 3.3	4.2 ± 1.4	1.000	0.889	**0.032**	0.143

Turicibacter	0	±	0.0	0	±	0.0	0	±	0.0	0	±	0.0	6.8	±	5.6	1.000	1.000	1.000	**0.032**
Brevibacterium	0	±	0.0	0	±	0.0	3.8	±	0.7	6.6	±	1.8	3.4	±	0.7	1.000	**0.032**	**0.032**	0.333
Streptococcus	6.2	±	0.0	1.2	±	0.8	1.8	±	0.5	2.2	±	0.8	1.2	±	0.5	0.143	0.143	0.167	**0.032**
Bhargavaea	0	±	0.0	0	±	0.0	3	±	1.4	6	±	2.2	2.2	±	1.0	1.000	**0.032**	**0.032**	0.095
Solibacillus	0	±	0.0	0	±	0.0	5.2	±	1.4	1.2	±	0.3	0.4	±	0.2	1.000	**0.032**	0.143	0.889
Candidatus Arthromitus	4.6	±	2.0	0.2	±	0.0	0	±	0.0	0	±	0.0	0	±	0.0	**0.032**	**0.032**	**0.032**	**0.032**

(d)

Genus in 12% group	day 0			day 7			day 14			day 21			day 28			Adjusted p values day 0 vs day 7	day 0 vs day 14	day 0 vs day 21	day 0 vs day 28
Lactococcus	0.25	±	0.2	1645	±	122.6	2119.4	±	66.2	1817.6	±	122.6	2012.4	±	91.5	**0.032**	**0.032**	**0.032**	**0.032**
Lactobacillus	1696.25	±	69.2	445	±	60.8	156.8	±	17.4	238	±	136.6	172.8	±	29.0	**0.032**	**0.032**	**0.032**	**0.032**
Bacteroides	177.75	±	36.9	75.8	±	26.2	51	±	22.4	107.6	±	67.8	22	±	5.1	0.095	0.095	0.151	**0.032**
Dorea	0	±	0.0	31.8	±	21.7	80.6	±	37.5	80.8	±	29.9	133.6	±	19.6	**0.032**	**0.032**	**0.032**	**0.032**
Citrobacter	0	±	0.0	66.2	±	52.3	21.4	±	18.8	130.2	±	46.2	59	±	35.8	**0.048**	**0.032**	**0.032**	**0.032**
Staphylococcus	128.75	±	23.9	47.4	±	4.7	7.8	±	2.0	6.8	±	1.2	2.4	±	0.4	**0.032**	**0.032**	**0.032**	**0.032**
Streptococcus	0	±	0.0	22.4	±	4.2	20.2	±	2.6	25.2	±	4.6	25.8	±	3.8	**0.032**	**0.032**	**0.032**	**0.032**
Atopostipes	5	±	1.5	13.6	±	6.1	9.6	±	1.1	0.2	±	0.2	0	±	0.0	0.302	0.302	3.000	**0.032**
Muribaculum	10.75	±	3.3	2.8	±	0.2	0.4	±	0.5	1.4	±	0.5	0.6	±	0.6	**0.032**	**0.032**	**0.032**	**0.032**
Enterococcus	0.25	±	0.2	4	±	4.6	6.8	±	3.1	9.4	±	3.6	10.4	±	4.0	**0.048**	**0.048**	**0.048**	**0.032**
Candidatus *Arthromitus*	5.25	±	1.2	0.6	±	0.0	0	±	0.0	0	±	0.0	0	±	0.0	**0.032**	**0.032**	**0.032**	**0.032**

Supplementary Table S2. The list of OTUs with more than 1% abundance in at least 1 group average in the intestinal microbiota of mice changed significantly (P < 0.05, Wilcoxon rank sum test with Benjamini-Hochberg p value correction) throughout the experiment. (a) The list of constitute species in 3% group at OTU level. (b) The list of constitute species in 12% group at OTU level with read numbers.

(a)

OTUs in 3% group	day0	day7	day14	day21	day28	day 0 vs day 7	vs day 14	vs day 21	vs day 28
OTU00001_Lactococcus lactis (100%)	0 ± 0.0	170.2 ± 52.9	151.6 ± 29.2	145.2 ± 8.8	115.6 ± 11.2	**0.032**	**0.032**	**0.032**	**0.032**
OTU00003_Staphylococcus lentus (100%)	292.2 ± 82.7	1652.2 ± 144.7	664.4 ± 46.9	500.2 ± 137.6	154.8 ± 17.8	**0.032**	**0.048**	0.619	0.619
OTU00002_Lactobacillus johnsonii (100%)	1290 ± 99.1	49 ± 42.7	0 ± 0.0	0.2 ± 0.2	0.4 ± 0.2	**0.032**	**0.032**	**0.032**	**0.032**
OTU00004_Citrobacter amalonaticus (100%)	83 ± 75.4	8.8 ± 4.2	522.4 ± 138.9	799.2 ± 121.0	708.4 ± 213.3	0.825	**0.048**	**0.032**	0.063
OTU00007_Enterococcus faecalis (100%)	0.2 ± 0.2	0 ± 0.0	0 ± 0.0	0 ± 0.0	667.4 ± 197.4	1.000	1.000	1.000	**0.032**
OTU00006_Jeotgalicoccus sp. L21-PYE-C15 (97.48%)	2.8 ± 0.4	515.6 ± 177.6	391.2 ± 26.5	120.2 ± 15.2	49.4 ± 13.9	**0.032**	**0.032**	**0.032**	**0.032**
OTU00005_Corynebacterium stationis (98.05%)	0 ± 0.0	0.4 ± 0.4	843 ± 23.8	1051.2 ± 31.6	567.6 ± 60.2	1.000	**0.032**	**0.032**	**0.032**
OTU00008_Lactobacillus murinus (100%)	162 ± 33.0	31.2 ± 15.2	35 ± 16.0	52.8 ± 27.0	290.2 ± 110.8	**0.032**	**0.032**	0.111	0.690

OTU00022_Staphylococcus nepalensis (100%)	46	±	15.3	230.4	±	85.9	11.4	±	3.3	2.2	±	0.4	0.8	±	0.6	**0.032**	**0.032**	**0.032**	**0.032**
OTU00015_Lactobacillus reuteri (100%)	282.4	±	45.3	2	±	1.4	0	±	0.0	0	±	0.0	0	±	0.0	**0.032**	**0.032**	**0.032**	**0.032**
OTU00009_Candidatus Dorea massiliensis (96.91%)	0.2	±	0.2	20.8	±	19.6	16.2	±	6.5	12	±	3.7	5.6	±	1.4	0.286	**0.048**	**0.048**	**0.032**
OTU00010_Bacteroides acidifaciens (99.68%)	55	±	9.4	16	±	14.0	8.8	±	3.6	7.8	±	4.0	13.8	±	12.8	0.095	**0.032**	**0.032**	0.095
OTU00027_Barnesiella viscericola (82.65%)	67.4	±	7.4	0	±	0.0	0	±	0.0	0	±	0.0	0	±	0.0	**0.032**	**0.032**	**0.032**	**0.032**
OTU00051_Enterobacter ludwigii (100%)	0	±	0.0	0	±	0.0	0	±	0.0	0	±	0.0	43.6	±	23.5	1.000	1.000	1.000	**0.032**
OTU00016_Porphyromonadaceae bacterium C941 (85.81%)	50.8	±	7.7	30.6	±	26.0	6.2	±	2.6	13.4	±	5.9	3.2	±	1.2	0.143	**0.032**	**0.032**	**0.032**
OTU00012_Rikenella microfusus (81.85%)	102.6	±	8.6	1.6	±	0.9	1	±	0.4	0.8	±	0.6	0.4	±	0.2	**0.032**	**0.032**	**0.032**	**0.032**
OTU00045_Acinetobacter sp. STM18 (100%)	0	±	0.0	0	±	0.0	0	±	0.0	0	±	0.0	63.4	±	19.7	1.000	1.000	1.000	**0.032**
OTU00033_Porphyromonadaceae bacterium C941 (86.77%)	75.6	±	17.5	0	±	0.0	0	±	0.0	0.2	±	0.2	0.2	±	0.2	**0.032**	**0.032**	**0.032**	**0.032**
OTU00030_Enterococcus gallinarum (100%)	0.6	±	0.4	0.2	±	0.2	0.2	±	0.2	33.2	±	20.9	65.6	±	11.8	1.000	1.000	0.310	**0.032**
OTU00018_Microbacterium	0	±	0.0	0	±	0.0	0	±	0.0	34.8	±	7.3	68.4	±	11.7	1.000	1.000	**0.032**	**0.032**

maritypicum (100%)									
OTU00017_Clostridiales bacterium CIEAF 017 (100%)	0.2 ± 0.2	10.4 ± 7.4	27.4 ± 4.3	0 ± 0.0	2.6 ± 1.5	0.889	**0.032**	1.000	0.500
OTU00019_Bacteroides caecimuris (99.37%)	34.8 ± 6.5	0.2 ± 0.2	0 ± 0.0	0.2 ± 0.2	0 ± 0.0	**0.032**	**0.032**	**0.032**	**0.032**
OTU00011_Lactococcus lactis (100%)	0 ± 0.0	3.4 ± 1.2	4.8 ± 1.9	3.8 ± 1.2	3.8 ± 1.2	0.095	**0.032**	**0.032**	0.095
OTU00023_Eubacterium coprostanoligenes (89.97%)	50.2 ± 8.0	0.6 ± 0.6	0 ± 0.0	0 ± 0.0	0 ± 0.0	**0.032**	**0.032**	**0.032**	**0.032**
OTU00031_Gabonia massiliensis (84.03%)	1.4 ± 7.9	0 ± 1.0	1.4 ± 0.0	0 ± 1.0	0 ± 0.0	**0.032**	**0.032**	**0.032**	**0.032**

(**b**)

	day0	day7	day14	day21	day28	day 0 vs day 7	vs day 14	vs day 21	vs day 28
OTU00001_Lactococcus lactis (100%)	0.2 ± 0.2	2010.0 ± 116.5	2038.2 ± 65.0	1706.2 ± 117.7	1996.8 ± 90.8	**0.032**	**0.032**	**0.032**	**0.032**
OTU00003_Staphylococcus lentus (100%)	113.6 ± 21.4	8.2 ± 2.0	3.2 ± 1.2	4.2 ± 0.7	0.0 ± 0.0	**0.032**	**0.032**	**0.032**	**0.032**
OTU00002_Lactobacillus johnsonii (100%)	1213.6 ± 99.1	20.6 ± 9.1	0.2 ± 0.2	0.4 ± 0.2	1.0 ± 0.4	**0.032**	**0.032**	**0.032**	**0.032**

OTU00004_Citrobacter amalonaticus (100%)	0.0 ± 0.0	67.8 ± 51.9	40.2 ± 18.4	112.8 ± 46.0	95.4 ± 35.5	**0.048**	**0.032**	**0.032**	**0.032**
OTU00022_Staphylococcus nepalensis (100%)	21.4 ± 4.5	6.8 ± 3.0	3.0 ± 0.9	1.6 ± 0.7	1.6 ± 0.2	0.056	**0.032**	**0.032**	**0.032**
OTU00015_Lactobacillus reuteri (100%)	197.8 ± 34.5	2.4 ± 0.7	0.0 ± 0.0	0.0 ± 0.0	0.0 ± 0.0	**0.032**	**0.032**	**0.032**	**0.032**
OTU00009_Candidatus Dorea massiliensis (96.91%)	0.0 ± 0.0	39.2 ± 21.7	89.4 ± 37.2	93.8 ± 29.6	120.4 ± 19.6	**0.032**	**0.032**	**0.032**	**0.032**
OTU00010_Bacteroides acidifaciens (99.68%)	71.6 ± 24.4	26.2 ± 14.2	35.0 ± 16.1	59.6 ± 34.9	13.2 ± 3.1	0.286	0.286	0.286	**0.032**
OTU00021_Lachnospiraceae bacterium 609 (99.07%)	0.4 ± 0.2	3.8 ± 2.0	21.4 ± 11.3	37.0 ± 12.2	62.0 ± 33.0	0.238	0.127	**0.032**	**0.032**
OTU00013_Longibaculum muris (86.75%)	1.0 ± 1.0	100.4 ± 32.0	77.2 ± 19.3	62.4 ± 16.1	62.0 ± 30.6	0.095	**0.032**	**0.048**	0.095
OTU00027_Barnesiella viscericola (82.65%)	79.0 ± 25.0	0.0 ± 0.0	0.2 ± 0.2	0.0 ± 0.0	0.0 ± 0.0	**0.032**	**0.032**	**0.032**	**0.032**
OTU00016_Porphyromonadaceae bacterium C941 (85.81%)	52.4 ± 16.6	32.0 ± 23.2	31.6 ± 11.8	15.2 ± 9.0	1.6 ± 0.7	0.333	0.333	0.095	**0.032**
OTU00012_Rikenella microfusus (81.85%)	66.0 ± 13.7	4.2 ± 2.4	4.0 ± 1.1	3.0 ± 1.0	1.6 ± 0.9	**0.032**	**0.032**	**0.032**	**0.032**
OTU00024_Streptococcus sp. oral clone AY020 (93.95%)	8.4 ± 1.1	42.8 ± 9.0	56.4 ± 7.8	61.0 ± 16.6	50.4 ± 7.1	**0.032**	**0.032**	**0.032**	**0.032**
OTU00033_Porphyromonadaceae	64.6 ± 15.1	1.6 ± 1.0	0.2 ± 0.2	0.8 ± 0.6	0.6 ± 0.4	**0.032**	**0.032**	**0.032**	**0.032**

bacterium C941 (86.77%)																		
OTU00030_Enterococcus gallinarum (100%)	0.2	±	0.2	9.4	±	4.6	4.6	±	2.9	7.4	±	3.6	14.4	±	4.0	**0.048**	**0.048**	**0.048**	**0.032**
OTU00029_Lachnospiraceae bacterium A4 (91.69%)	0.8	±	0.8	13.2	±	6.6	19.6	±	3.3	47.8	±	19.9	54.6	±	12.0	0.087	**0.032**	**0.032**	**0.032**
OTU00017_Clostridiales bacterium CIEAF 017 (100%)	0.0	±	0.0	20.8	±	12.9	46.8	±	13.3	58.4	±	15.1	24.4	±	3.7	0.444	**0.032**	**0.032**	**0.032**
OTU00044_Lachnospiraceae bacterium A4 (98.72%)	30.6	±	16.8	0.0	±	0.0	0.0	±	0.0	0.0	±	0.0	0.0	±	0.0	**0.032**	**0.032**	**0.032**	**0.032**
OTU00019_Bacteroides caecimuris (99.37%)	61.0	±	8.4	1.0	±	0.4	0.2	±	0.2	0.4	±	0.2	0.0	±	0.0	**0.032**	**0.032**	**0.032**	**0.032**
OTU00011_Lactococcus lactis (100%)	0.0	±	0.0	57.8	±	6.7	59.4	±	4.9	55.0	±	5.6	65.4	±	3.8	**0.032**	**0.032**	**0.032**	**0.032**
OTU00023_Eubacterium coprostanoligenes (89.97%)	32.6	±	9.3	0.6	±	0.2	0.0	±	0.0	0.0	±	0.0	0.0	±	0.0	**0.032**	**0.032**	**0.032**	**0.032**
OTU00031_Gabonia massiliensis (84.03%)	3.4	±	6.4	0.2	±	2.2	0.0	±	0.2	0.0	±	0.0	0.0	±	0.0	**0.032**	**0.032**	**0.032**	**0.032**

Supplementary Table S3. Nutrient composition of the 3%, 6%, 9%, and 12% protein diets used in this study. Raw material mixture and estimated nutrient contents per 100 g are listed.

	3%	6%	9%	12%
Raw material mixture (g)				
Milk caseins	3.3	6.7	10.0	13.3
L-cysteine	0.05	0.10	0.15	0.20
Cornstarch	52.4	49.8	47.3	44.8
α-cornstarch	17.5	16.6	15.8	14.9
Sucrose	10.0	10.0	10.0	10.0
Soybean oil	7.0	7.0	7.0	7.0
Cellulose powder	5.0	5.0	5.0	5.0
AIN-93G mineral mixed	3.5	3.5	3.5	3.5
AIN-93 vitamin mixed	1.0	1.0	1.0	1.0
Choline bitartrate proportion	0.25	0.25	0.25	0.25
Tertiary butyl hydroquinone	0.0014	0.0014	0.0014	0.0014
Estimated nutrients composition (%) and energy content (kcal)in 100g diet				
Crude protein	3.0	6.0	9.0	12.0
Crude fat	7.0	7.0	7.0	7.0
Nitrogen free extract	81.6	78.2	74.8	71.5
Energy	401	400	398	397

Supplementary Table S4. Sample size estimation to power our study with body weight data based on significance level 0.05 and statistical power 0.80.

Day	Significance level	Power	SE	SD	Average body weight				Sample size
					3%	6%	9%	12%	
0	0.05	0.80	0.62	1.38	27.8	28.0	27.9	27.9	2558
7	0.05	0.80	0.76	1.70	27.4	28.8	29.2	30.0	39
14	0.05	0.80	0.81	1.81	26.2	29.1	29.8	31.0	16
21	0.05	0.80	0.85	1.91	26.8	30.0	30.4	32.1	16
28	0.05	0.80	0.87	1.95	26.7	30.2	31.0	32.1	15
